# Characterization and Initial Optimization of Reconstruction Performance for Detector Crosstalk of Ultrahigh Performance Brain PET Imager

**DOI:** 10.1109/trpms.2026.3698769

**Published:** 2026-06-01

**Authors:** Jiazhen Zhang, Kathryn Fontaine, Tiantian Li, Paul Gravel, Takuya Toyonaga, Yifan Hu, Jean-Dominique Gallezot, Tianyi Zeng, Tommaso Volpi, Yihuan Lu, Thibault Marin, Jinyi Qi, Richard E. Carson

**Affiliations:** Department of Biomedical Engineering, Yale University, New Haven, CT 06519 USA; Department of Radiology and Biomedical Imaging, Yale University, New Haven, CT 06519 USA; United Imaging Healthcare America, Houston, TX 77054 USA; Department of Radiology and Biomedical Imaging, Yale University, New Haven, CT 06519 USA; Department of Radiology and Biomedical Imaging, Yale University, New Haven, CT 06519 USA; Department of Radiology and Biomedical Imaging, Yale University, New Haven, CT 06519 USA; Department of Radiology and Biomedical Imaging, Yale University, New Haven, CT 06519 USA; Department of Radiology and Biomedical Imaging, Yale University, New Haven, CT 06519 USA; Department of Radiology and Biomedical Imaging, Yale University, New Haven, CT 06519 USA; United Imaging Healthcare, Shanghai 201807, China; Department of Radiology and Biomedical Imaging, Yale University, New Haven, CT 06519 USA; Department of Biomedical Engineering, University of California, Davis, Davis, CA 95616 USA; Department of Radiology and Biomedical Imaging and the Department of Biomedical Engineering, Yale University, New Haven, CT 06519 USA

**Keywords:** Detector crosstalk, high resolution, image reconstruction, NeuroEXPLORER (NX), positron emission tomography (PET), point spread function (PSF)

## Abstract

The NeuroEXPLORER (NX) is a next-generation, ultrahigh-performance brain positron emission tomography/computed tomography (PET/CT) system designed for both high spatial resolution and sensitivity. To meet high-sensitivity (HS) requirements, the system uses 2-cm-long scintillation crystals, while the novel U-shaped depth-of-interaction (DOI) encoding and small crystal elements enable high resolution. However, using long, small crystals increases signal crosstalk between crystals arising from Compton scattering, photoelectron escape, and incomplete optical isolation, which can degrade spatial resolution. Although the NX electronics detect crosstalk between microblocks and support real-time recovery using measured energy, accurate identification of the first interaction point is not always possible. Incorporating crosstalk events increases coincidence sensitivity by approximately 50% but compromises spatial resolution. Given such a large fraction of detector crosstalk, evaluating its impact on reconstruction performance in the NX is imperative. In this work, we evaluated image resolution in three reconstruction modes: (HS; all events), higher resolution (HR; noncrosstalk events), and lower resolution (LR; crosstalk events), using real phantoms and human studies with multiple tracers. Guided by the resolution characterization of the HYDRA-OLG brain phantom, we introduced a two-Gaussian mixture point spread function (2G-PSF) for HS reconstruction to improve contrast while suppressing noise. At matched iterations, HR yielded the highest image contrast, HS showed slightly reduced contrast, and LR exhibited the largest partial volume effect, as expected. Modeling crosstalk with the 2G-PSF for HS increased contrast toward HR levels without sacrificing sensitivity. Overall, these results provide a practical strategy for including crosstalk events in high-resolution PET systems while mitigating their impact on spatial resolution.

## I. Introduction

The NeuroEXPLORER (NX) [1], [2], [3], a brain-dedicated positron emission tomography/computed tomography (PET/CT) scanner, has been designed and built with high spatial resolution and high sensitivity. Its design includes a long axial coverage (49.5 cm) to achieve high sensitivity and a novel U-shaped depth-of-interaction (DOI) detector with small crystals in the transaxial direction to achieve high spatial resolution. As a result, the system’s performance can be substantially influenced by detector crosstalk, i.e., when a single incident gamma event causes signals in more than one readout channel [4], which can consequently degrade spatial resolution. Detector crosstalk arises from Compton scattering from one crystal to another [5], [6], [7], [8], [9], or the escape of the photoelectron from the primary crystal following photoelectric absorption [10]. In addition, optical isolation of the scintillation photons within a single crystal is never absolute, also leading to crosstalk [11].

Gamma-ray interactions in matter are well modeled in commonly used simulation toolkits [12], [13]. Several studies have previously investigated Compton crosstalk associated with Compton scattering, and its impact on the system’s performance and spatial resolution, using both experiments and simulations. Vaska et al. [7] identified the primary crystal as the channel with the largest energy and recovered crosstalk energy by summing neighboring signals, deriving spatial resolution from flood-map peak separation. Shao et al. [5] used Monte Carlo simulations (MCSs) to record all interaction sites and deposited energies, assigned the event to a crystal using one of several positioning rules, and then quantified resolution with coincidence line-spread functions (CLSFs) using two opposing small-pixel detector arrays. Stickel and Cherry [8] modeled intrinsic resolution physics, including intercrystal scatter (ICS), and analyzed the projection-space modulation transfer function (MTF) using MCS without event-level corrections. Zeraatkar et al. [14] also used MCS to separate coincidences with/without ICS and built class-specific system response functions (SRFs) in projection space. On the contrary, Tohme and Qi [15] directly estimated the detector blurring component (including ICS) [16] of the system matrix for iterative image reconstruction from point source measurements. More recently, Petersen et al. [17] employed both a statistical Bayesian estimation framework and deep neural networks to estimate interaction coordinates, including deposited energies and DOIs, in the two nearest-neighbor crystals involved in an ICS event, and then identified the initial interaction point. Lee et al. [18] reviewed various methods, including energy-based, Compton kinematics-based, statistical, and AI approaches, developed to recover ICS events. Most recently, Kuang et al. [19] implemented a method to identify crosstalk events by analysis of the flood histograms on the SIAT aPET, a small-animal scanner, and demonstrated the impact on resolution loss using phantom data. Their study, however, did not explore the potential for leveraging these crosstalk events.

Optical crosstalk associated with imperfect optical isolation also negatively affects positioning accuracy. Both photoelectric absorption and Compton scattering of gamma photons deposit energy in scintillation detectors, producing scintillation (optical) photons. These optical photons can propagate within the scintillator and into neighboring detector elements, causing optical crosstalk, which is of great interest and still under investigation [11]. Meanwhile, optical MCS has been developed to study the behavior of optical photons. Optical photon reflections and transmissions through the interfaces were modeled using the lookup table (LUT) Davis model [20] and validated by real experiments [21], [22].

The NX was designed to directly detect intermicroblock crosstalk (IBC) (see Fig. 1) and perform real-time recovery using gamma-photon energy: energy signals are summed for each microblock and its neighbors, and the location with the highest deposited energy is assigned as the initial interaction point [23]. As a result, each of the two singles in a coincidence is assigned a one-bit flag in the list-mode data, indicating whether it has been identified as IBC-recovered (1) or unaffected by crosstalk (0). In practice, incorporating IBC events increases coincidence sensitivity by approximately 50% (with the number of IBC events being about half that of non-IBC events), but it can degrade spatial resolution if the initial interaction point is misassigned [24]. Given the substantial IBC fraction, a dedicated framework to characterize and account for IBC in the NX using real experimental data is extremely desirable and may also guide future high-resolution systems.

In this work, we characterized how IBC affects image resolution on the NX using three reconstruction modes: high sensitivity (HS; including all events), higher resolution (HR; including events in which neither detector was labeled as IBC), and lower resolution (LR; including events in which one or both detectors were labeled as IBC). Here, we relied exclusively on real phantom and human data to capture the system’s true response. To account for the effect of IBC on spatial resolution, we introduced a two-Gaussian mixture point spread function (2G-PSF) for HS reconstruction (HS-2G) to improve image contrast while suppressing noise. We evaluated the proposed 2G-PSF on miniDerenzo and high-resolution brain phantoms, as well as human scans with four different tracers.

## II. Materials and Methods

### A. Detector Crosstalk in the NX

The NX [1] consists of 20 detector modules forming a cylindrical detector ring with a diameter and axial field of view (FOV) of 52.4 and 49.5 cm, respectively. Each module contains six NX blocks aligned along the axial direction, except for six modules in the shoulder-cutout region, where one axial block is removed (leaving five blocks per module) to accommodate subject positioning. Each NX detector block consists of a 12 × 12 array of microblocks, where each microblock consists of four U-shaped light-sharing paired detectors. Each U-shaped element comprises two lutetium-yttrium oxyorthosilicate (LYSO) crystals measuring 1.56 × 3.07 × 20.0 mm^3^, with a single-ended readout by SiPMs (see Fig. 1). Consequently, each microblock is a 4 × 2 array of crystals read out by a 2 × 2 array of 3-mm SiPMs.

The NX electronics identifies IBC in real time by summing energy signals from each microblock and its neighbors. If the sum of the two highest energy signals exceeds the lower level discriminator (LLD, 430 keV) and each of those two signals exceeds the IBC raw cut (160 keV), the crystal with the higher deposited energy is assigned as the most likely initial interaction point [23]. This logic is performed within field-programmable gate arrays (FPGAs), with signals from each NX block analyzed by two FPGAs. Consequently, only microblocks managed by the same FPGA can participate in the IBC calculation. For each coincidence, the corresponding two singles are each assigned a one-bit flag in the list-mode data. A value of 1 denotes that the single is IBC-recovered, while 0 indicates no crosstalk. Using this event-specific IBC information, list-mode reconstruction with MOLAR-NX [25] can operate in three different modes.

1. *HS Mode:* Uses all collected events.
2. *HR Mode:* Uses only events where neither photon was marked as IBC recovered.
3. *LR Mode:* Uses events where one or both photons were marked as IBC recovered.

### B. Datasets

#### 1) Phantom Studies:

A 0.25-mm ^22^Na point source (~1 MBq) was scanned for 2 min at three different locations. The source was positioned at the center of the scanner (position 1), 8 cm transaxially off-center along the *y*-axis (position 2), and 12 cm axially off-center (position 3).

A miniDerenzo phantom (filled with ^18^F-FDG solution of ~71 MBq) was scanned at the center of the system for 10 min. The phantom consists of six sectors containing radioactive rods with varying diameters (1.2, 1.6, 2.4, 3.2, 4.0, and 4.8 mm), with the center-to-center distance between the rods being twice their diameter. The phantom was oriented so that the rods were parallel to the axial direction of the scanner.

A HYDrodynamically filled Realistic Anatomy of the Occipital Lobe, Gray matter only (HYDRA-OLG) phantom [26], [27] (filled with ^18^F-FDG solution of ~11 MBq) was scanned at the center of the system for 25 min. This phantom was 3-D-printed based on the BigBrain atlas [28], [29] and is designed to better represent the fine structures of the human brain, targeted for use with high-performance scanners such as the NX.

#### 2) Human Studies:

Five healthy human subjects (3M/2F, 48 ± 8 yo) participated in IRB-approved protocols, including four tracers: ^18^F-FDG (n = 2; glucose metabolism), ^18^F-SynVesT-1 (n = 1; synaptic protein SV2A, a marker of synaptic density [30]), ^18^F-FPEB (n = 1; mGluR5 metabotropic glutamate receptors [31]), and ^18^F-Flubatine (n = 1; *α*4β2* nicotinic acetylcholine receptors [32]). The first three tracers have high uptake across gray matter (GM), whereas the last tracer displays focal uptake in the subcortex and brainstem [e.g., inferior olivary nuclei (ION)]. ^18^F-FDG (327 and 370 MBq), ^18^F-SynVesT-1 (177 MBq), ^18^F-FPEB (156 MBq), and ^18^F-Flubatine (169 MBq) were administered in a bolus+infusion protocol (bolus duration = 1 min, *K*_bol_ = 90, 150, 190, and 360 min, respectively). List-mode data were acquired for 90 min for ^18^F-FDG and 120 min for the other tracers. Magnetic resonance imaging (MRI) was performed on a 3T Prisma scanner (Siemens Medical Systems, Erlangen, Germany), producing T1-weighted (T1w) images for brain parcellation with 1.0-mm isotropic voxels. Written informed consent was obtained from all participating subjects.

### C. Image Reconstruction

All images were reconstructed using MOLAR-NX [25] with the following parameters and corrections: 3-D ordered-subsets expectation-maximization (OSEM) algorithm (1–20 iterations, five subsets) with 0.5-mm isotropic voxels, attenuation estimated from CT, randoms estimated from detector block singles rates, time-of-flight (TOF), DOI, single scatter simulation (SSS) with relative scaling and scatter estimates distributed into TOF space weighted by the distribution of prompts minus delays [33], normalization using components provided by United Imaging Healthcare (UIH), dead-time correction, and vendor suggested, spatially invariant Gaussian image-based point spread function (PSF) [34] with a full width at half maximum (FWHM) of 1.4 × 1.4 × 2.0 mm^3^ (denoted 1G-PSF). Unless otherwise noted, this 1G-PSF resolution model was used for all reconstructions (HR, LR, and HS); the only exception is HS-2G, which uses the 2G-PSF described in Section II-F. Event-by-event motion correction (MC) was also performed for human studies with motion information acquired by the UIH markerless motion tracking (UMT) system [35], [36], [37], [38].

For the point source reconstruction, only an antialiasing filter (0.9 × 0.9 × 1.6 mm^3^ FWHM) was applied instead of the 1G-PSF. In addition, 2-D filtered backprojection (FBP) reconstructions using a ramp filter with 0.5-mm isotropic voxels and without attenuation correction, scatter correction, TOF, or postsmoothing were performed for comparison.

Each phantom and human dataset was reconstructed using different subsets of events grouped by event type to produce the HS-, HR-, and LR-mode images. Human time windows [3] were 60–90 min postinjection for ^18^F-FDG, ^18^F-SynVesT-1, and ^18^F-FPEB, and 60–120 min postinjection for ^18^F-Flubatine. For the HYDRA-OLG, we also generated count-matched HS and HR frames, shortened to match LR statistics for fair comparisons.

### D. Normalization and Quantitation

To maintain quantitative consistency and avoid normalization-induced artifacts across modes, we applied: 1) a global scaling factor derived from the HS-to-HR and HS-to-LR prompts-minus-delays ratios. Empirically, these ratios were stable across count levels, supporting a consistent global scaling and 2) mode-specific normalization factors for HS, HR, and LR. In particular, the component-based normalization was produced using data from a cylindrical uniform phantom filled with ^18^F-FDG solution, which was placed at the center of the FOV. The non-IBC events and IBC events from the same normalization scan were used to generate normalization components for the HR and LR modes, respectively.

### E. Characterization of Spatial Resolution

We assessed spatial resolution across modes using both phantom and human data as a function of OSEM iteration. For point sources, FBP and OSEM reconstructions were fit with an eight-parameter 3-D Gaussian function to estimate in-plane (x, y) and axial (z) FWHM. In particular, the 3-D Gaussian function included parameters for the three centroid positions, three FWHMs, an amplitude term, and a constant background offset. The fit was performed over a small 3-D volume (~10-mm cube) centered on each point source to capture the local point-spread distribution. While useful for characterizing intrinsic system resolution, these point-source estimates do not generally reflect the resolution of reconstructed images in practice [39], [40]. To better approximate in vivo conditions, we estimated resolution with the HYDRA-OLG phantom by fitting each PET image to a CT-derived binary reference image convolved with a 3-D Gaussian filter, selecting the filter parameters that minimized a least-squares cost *J*(·)

(1) $$J\left(k\right)={\left\|r*k-x\right\|}_{2}^{2}$$

where *r* is the binary reference image, *k* is a 3-D Gaussian kernel with separate in-plane and axial smoothing, * is the convolution operator, and *x* is the reconstructed PET image. Optimization used a Levenberg–Marquardt solver. In addition, linear terms (amplitude and background) were also included to accommodate global intensity differences. Fits were performed for the HS, HR, and LR modes, including count-matched frames for HS and HR to assess noise sensitivity. As shown in Fig. 2, the binary reference image was created by subtracting the high-resolution CT images of the filled and empty phantoms, which were coregistered to the same PET reconstruction, and thresholded to match the phantom volume.

In addition to Gaussian FWHM-based resolution metrics, we also computed the peak-to-valley ratio (PVR) from miniDerenzo rod profiles to directly compare image contrast across modes as a function of OSEM iteration. The PVR was computed by identifying all peak and valley intensities along the rod profile and taking the ratio of mean peak values to mean valley values. Similarly, we present intensity profiles from the HYDRA-OLG phantom and human images over targeted regions of interest (ROIs) for quantitative comparison. For the intensity profile analysis, we assessed not only the HS, HR, and LR reconstructions, but also an optimized HS reconstruction incorporating a 2G-PSF model (HS-2G), as described in Section II-F. To distinguish these configurations, only the HS reconstruction using the standard single-Gaussian PSF is denoted as HS-1G, while the HR and LR reconstructions both employ the same 1G-PSF model. Representative slices for the four modes are also shown for qualitative comparison.

### F. Initial Reconstruction Optimization for Detector Crosstalk

Guided by the HYDRA-OLG fits in Section II-E, we propose parameterizing the PSF of HS as a two-component Gaussian mixture (2G-PSF) to improve image quality. This approach provides a simple initial optimization relative to event-specific multi-PSF modeling, which would require estimating event-dependent normalization terms and mode-dependent scaling factors for sensitivity maps within the iterative reconstruction, thereby increasing implementation complexity and computational cost. The 2G-PSF is defined as

(2) $$\Psi =\sum_{j=1}^{2}w_{j}G_{j}$$

where *G*_*j*_ denotes the PSF kernel for event type *j* and *w*_*j*_ is its corresponding proportion $\left(\sum_{j}w_{j}=1\right)$. Based on the statistics of list-mode data collected on the NX, non-IBC events comprise ~ 63.4%(±0.2%) (*w*_1_) and IBC events ~ 36.6%(±0.2%) (*w*_2_). We set *G*_1_ to the standard PSF (1G-PSF), i.e., a 1.4 × 1.4 × 2.0 mm^3^ FWHM Gaussian, representing non-IBC events, and *G*_2_ is a broader kernel representing IBC events.

Using the measured HR–LR resolution gap, we defined a Gaussian kernel *G*_diff_ with

(3) $${\text{FWHM}}_{\text{diff}}=\sqrt{{\text{FWHM}}_{\text{LR}}^{2}-{\text{FWHM}}_{\text{LR}}^{2}}$$

where FWHM_LR_ and FWHM_HR_ are obtained from the fit Gaussian filter *k* for LR and HR, respectively. We then set

(4) $$G_{2}=G_{1}*G_{\text{diff}}$$

so that, by Gaussian variance additivity under convolution, the effective FWHM of *G*_2_ is

(5) $${\text{FWHM}}_{2}=\sqrt{{\text{FWHM}}_{1}^{2}+{\text{FWHM}}_{\text{diff}}^{2}}$$

where FWHM_1_ is the FWHM of *G*_1_.

### G. Noise Evaluation

We assessed the noise properties of the four reconstruction modes (HS-1G, HS-2G, HR, LR) as a function of OSEM iteration using human data. Using the T1w MR images, volumetric segmentation was performed with FreeSurfer [41] (version 6.0.0), which segmented each individual’s brain into 109 ROIs. All GM ROIs were merged into one large ROI as total GM (TGM). PET list-mode data were uniformly subsampled by timestamp into two interleaved frames with equal counts to examine noise properties. We reconstructed both frames, formed a difference image *d* (frame 2 minus frame 1), and extracted voxel values within TGM. This approach suppresses biological heterogeneity within GM, so the residual variation reflects image noise. We then calculated the coefficient of variation (CoV) as a function of iteration for each mode

(6) $$\text{CoV}=\frac{\sigma }{\mu }=\frac{\sqrt{\frac{1}{n-1}\sum_{i\in \text{TGM}}{\left(d_{i}-\overset{-}{d}\right)}^{2}}}{\frac{1}{n}\sum_{i\in \text{TGM}}x_{i}}$$

where *x* is the full-count reconstructed image, *d* is the difference image between two replicates, *n* is the number of voxels in TGM, and d-=1n∑i∈TGMdi.

### H. Additional Validation

Although this work focuses on spatial-resolution characterization and optimization of the HS mode, we also performed two additional evaluations using one ^18^F-FDG study. First, we assessed quantitative consistency across modes. As described in Sections II-C and II-D, by performing mode-specific normalization, random correction with detector block singles rates, scatter correction using SSS, and scaling by the prompt-minus-delay ratios, the reconstructed HR and LR images provide quantitative activity estimates. We performed ROI quantification for FreeSurfer-segmented gray-matter ROIs for each mode. Second, we evaluated an updated 1G-PSF for LR. The vendor-suggested 1G-PSF model was derived from an MCS that does not explicitly account for detector crosstalk; therefore, the standard 1G-PSF (*G*_1_ = 1.4 × 1.4 × 2.0 mm^3^) is most appropriate for HR data. Using the HR-to-LR resolution difference quantified in Section III-C, we derived a corresponding widened Gaussian component (*G*_2_) for LR (LR-w1G) via (4), which was used for a subset of the results shown and discussed in Section III-D1.

## III. Results

### A. Point Sources

Fig. 3 shows the fit FWHM in the *x*, *y*, and *z* directions for HS, HR, and LR as a function of OSEM iteration (2–20), with FBP shown at the left. Across all three positions, HR consistently yields the best FWHM, LR the poorest, with HS in between. OSEM with PSF modeling sharpens all modes relative to FBP. HS appears similar to HR because OSEM’s nonlinearity and the nonnegativity constraint can artificially sharpen isolated point sources lacking background. The distinction between the two modes becomes more pronounced in the more complex brain phantom (see Section III-C).

### B. MiniDerenzo Phantom

Fig. 4 shows the 20th-iteration reconstructions of the miniDerenzo phantom for each mode. Radial and tangential line profiles through the 2.4-mm rods are used to compute the PVR. HR achieves the highest PVR and best rod resolvability, outperforming HS-1G and LR. Consistent behavior is observed across different rod sizes.

Fig. 5 shows PVR as a function of OSEM iteration (1–20) for the 2.4-mm rods. HR (orange) achieves the highest PVR, exceeding HS-1G (blue) and LR (yellow). These three curves trended toward convergence. HS-2G results are discussed in Section III-C.

### C. HYDRA-OLG Phantom and Estimation of 2G-PSF

Fig. 6 shows the fit Gaussian FWHM in the transaxial and axial directions as a function of OSEM iteration (3–20) for HYDRA-OLG reconstructions across modes. Because LR had fewer counts than HR and HS, we subsampled HS and HR to match the LR statistics. Subsampled HS and HR yielded nearly identical FWHMs to the full-count results, indicating robustness to noise. Nonlinear fits to the CT-derived reference image revealed mode-dependent blur: HR required the least amount of smoothing, LR the most, and HS was in between. The between-mode differences in axial blur were smaller because the 1G-PSF uses a larger axial FWHM. With increasing iterations, HR converged to a similar transaxial and axial FWHM, suggesting that the 1G-PSF describes non-IBC data well.

The relatively constant HR-to-LR FWHM difference across iterations provided the empirical input for *G*_2_ in the HS 2G-PSF model (see Section II-F). Averaging the HR-to-LR resolution difference calculated via (3) over the 11th to 20th iterations yielded FWHM_diff_ to be 2.36 mm (in-plane) and 2.12 mm (axial), giving *G*_2_ to be a 2.74 × 2.74 × 2.92 mm^3^ FWHM Gaussian kernel. The 2G-PSF was then computed via (2) and applied to all datasets except the point sources.

As shown in Fig. 4, HS-2G nearly matches the HR tangential and radial profiles. In Fig. 5, HS-2G approaches the HR curve with increasing iterations, indicating that the 2G-PSF requires more iterations to converge. HS-2G surpasses HS-1G at the 11th iteration (tangential) and the 15th iteration (radial), corresponding to approximately 55 and 75 image updates (iterations × subsets), respectively, comparable to our standard OSEM setting (7 iterations and 10 subsets).

Fig. 7 shows 20th-iteration reconstructions of the HYDRA-OLG phantom alongside the reference image and a selected transverse line profile. The profiles indicate that HR achieves a higher PVR (9.4) than HS-1G (7.1) and LR (4.3). HS-2G closely matches HR in both appearance and profile contrast (9.0).

### D. Human Studies

Here, we present representative slices with line profiles drawn across small, focal-uptake structures [e.g., inferior colliculi (IC), ION, and so on] and across cortical gray-matter folds. We also include the noise analysis described in Section II-G and the additional validation described in Section II-H, applied to one ^18^F-FDG study.

#### 1) ^18^F-FDG:

In Fig. 8, coronal slices from the 20th-iteration OSEM reconstructions for two subjects imaged with ^18^F-FDG show that the HR images provide the sharpest delineation of GM relative to HS-1G and LR. Line profiles through the (IC; ~4 mm in diameter) confirm that HR achieves the highest peak-to-valley contrast. Our proposed HS-2G closely matches this contrast and exhibits the lowest image noise among all methods based on both visual assessment and quantitative noise evaluation (see below). LR also reproduces the two-peak IC structure but with broadened peaks and reduced contrast. Overall, incorporating 2G-PSF in HS mode improves contrast toward HR without discarding counts.

Percent differences (%) in ROI quantification relative to HS-1G (reference) were computed for each mode at iteration 20 across 11 FreeSurfer-defined gray-matter ROIs spanning cortical, subcortical, and cerebellar regions. LR showed a consistently negative bias (−3.24% ± 1.35%), due to stronger partial volume effects, whereas HR showed a positive bias (1.49% ± 0.66%), indicative of reduced partial volume loss. HS-2G also exhibited a positive bias (0.74% ± 0.49%) consistent with improved recovery enabled by the 2G-PSF model. Overall, the observed differences are small, indicating good quantitative agreement across modes.

For ^18^F-FDG Subject 1, noise was evaluated using the CoV defined in (6). Fig. 9(a) shows CoV as a function of OSEM iteration (2–20) for each mode. Using the same standard 1G-PSF, HS-1G yielded a lower CoV than HR and LR because it has the highest counts, and HS-2G further reduced CoV relative to HS-1G. Fig. 9(b) illustrates the PVR of IC as a function of CoV across OSEM iterations (2–20). At the 20th iteration, HS-2G demonstrates improvements in both PVR and CoV (10.0 and 0.23, respectively) compared to HS-1G (9.0 and 0.29). Owing to its slower convergence, HS-2G also exhibits lower noise compared with HR at the 20th iteration (PVR: 10.6 and CoV: 0.33). If additional iterations were performed, HS-2G would presumably approach the performance of HR while keeping a low noise level, as the current PVR trend has not yet plateaued. At a matched CoV of about 0.23, HS-2G attains a PVR of 10.0, whereas HR reaches a PVR of 9.0. Applying the widened 1G-PSF to LR [LR-w1G, using *G*_2_ derived in (4)] improves both CoV (0.21) and PVR (6.9) relative to LR with standard 1G-PSF (CoV: 0.43, PVR: 6.5) at the 20th iteration. However, even at a matched CoV of 0.21, LR-w1G still yields lower PVR than HS-1G, HR, and HS-2G.

#### 2) ^18^F-SynVesT-1:

Fig. 10 shows reconstructed images with intensity profiles drawn across two different thalamic nuclei: the medial geniculate nuclei (~4 mm in diameter) on a transverse slice (a), and the anterior thalamic nuclei (~4 mm in diameter) on a coronal slice (b). HR again achieves the highest PVR, outperforming HS-1G and LR. Incorporating the 2G-PSF in HS mode improves the profiles relative to HS-1G and reduces noise. In the profile of the medial geniculate nuclei, LR diverges from the HS and HR trends, consistent with its lower resolution.

#### 3) ^18^F-FPEB:

Fig. 11 shows coronal slices with intensity profiles drawn across the opercular cortex and the olfactory bulb (~3 mm in diameter). In the opercular cortex, the right-hand peak appears at slightly different positions across modes. This variability likely arises from count-level differences (noise) and mode-dependent spatial resolution interacting with the complex cortical anatomy. We did not observe this effect in the point-source or miniDerenzo phantoms, which have simpler structures and no background activity. In the olfactory bulb, the LR profile was considerably flattened, consistent with its lower contrast and higher noise due to limited counts.

#### 4) ^18^F-Flubatine:

Fig. 12 shows transverse slices with intensity profiles drawn across the pituitary gland and the (ION; ~5 mm in diameter). Although this tracer yields limited gray/white-matter contrast in static images, uptake is relatively uniform, and HS-2G consistently exhibits the lowest noise in slices based on visual assessment. For focal uptake, HR maintains the highest contrast, whereas LR shows greater variability, consistent with higher noise.

Table I summarizes the PVR values of all selected profiles across subjects for each mode. Overall, the target-ROI contrast was highest for HR and HS-2G, followed by HS-1G, with LR exhibiting the lowest contrast and the most substantial partial volume effects into adjacent tissue.

For cortically distributed tracers (^18^F-FDG, ^18^F-SynVesT-1), HR yielded the sharpest gray-matter delineation and the highest small-structure-to-background contrast. HS was similar but slightly blurred, and LR exhibited the largest partial volume effects. Replacing the 1G-PSF with the 2G-PSF in HS reconstructions improves contrast in human images while suppressing noise relative to HR. Visual assessment also shows HS-2G approaching HR-level contrast across many structures while preserving HS sensitivity.

## IV. Discussion

We characterized and optimized reconstruction performance for detector crosstalk in a next-generation brain PET system, the NX. To the best of our knowledge, this is the first study to characterize and optimize detector crosstalk for reconstruction performance using both a next-generation 3-D high-resolution brain phantom designed for ultrahigh-performance PET systems and real human PET data. The high resolution of the NX and the use of tracers with focal uptake in human scans enable the assessment of resolution in vivo and construct measures similar to those obtained from phantoms. The resulting framework serves as a practical and broadly applicable recipe for evaluating, comparing, and improving the performance of modern high-resolution scanners. By strategically exploiting detector crosstalk events, this approach improves image contrast and reduces noise. Both characterization and optimization are possible by the identification of crosstalk events in the NX list-mode data, which is crucial for modern systems, as detector crosstalk events can be rejected or retained and explicitly modeled to improve image contrast depending on the application.

Patient motion during PET acquisition blurs images and biases tracer concentration estimates. The high-resolution capability of the NX (or any high-performance PET system) can be realized only with accurate MC. In the human studies presented in this work, motion estimates were considered to be reliable, as indicated by smoothly varying brain motion-corrected centroid-of-distribution (MC-COD) [42], [43] curves with UMT motion information.

Here, we fit point source data acquired at multiple spatial positions, spanning the center of the FOV to more peripheral locations representative of the human brain, using a 3-D Gaussian model to characterize the system’s PSF and to enable direct comparisons across acquisition modes. However, point sources alone are inadequate for estimating true resolution differences because OSEM is nonlinear, and the nonnegativity constraint can artificially sharpen isolated point reconstructions lacking background. As a result, HS-1G provided very similar results to HR (see Fig. 3). Meanwhile, a single-Gaussian fit under-parameterized HS, which contains non-IBC and IBC events at approximately a 2:1 ratio. To better reflect human brain conditions, we therefore first quantified resolution using the high-resolution brain phantom. Not surprisingly, larger differences between HS-1G and HR were observed with not only the high-resolution brain phantom but also the human data. Since HS consists of HR and LR events, a two-Gaussian mixture would more faithfully represent the PSF of HS. For fairness and comparability across modes, we also reported the HS resolution using a single Gaussian. This choice also simplifies the derivation of the HR–LR resolution gap, which we then used to model HS as a two-Gaussian mixture, i.e., 2G-PSF.

Introducing a second Gaussian in the 2G-PSF increases the effective PSF kernel width. In iterative reconstruction, a broader kernel generally slows convergence [44], consistent with the behavior observed in Fig. 5. Despite this slower convergence, HS-2G still provided a clear improvement over HS-1G after only 100-image updates (20 iterations ×5 subsets) for both phantom and human studies. For finer resolution features, however, additional iterations may be required. This behavior is reflected in the 1.6-mm rods of the miniDerenzo phantom: HS-2G exhibited a lower PVR than HS-1G at the 20th iteration (1.8 versus 2.1), whereas they achieved comparable PVRs by the 60th iteration (3.6 versus 3.5). On the contrary, the larger 2.4-mm rods shown here converge more rapidly than smaller ones. To ensure a fair comparison, we matched the iteration number across modes and avoided over-iteration, as excessive iterations introduce not only higher noise but also noise-induced bias [45].

To further enhance image quality, reconstruction parameters (e.g., number of OSEM iterations and subsets) should be optimized [46] for specific applications, as different resolution features and contrast levels exhibit distinct convergence behaviors. This consideration is especially important for in vivo imaging tasks involving small structures. For instance, the left and right dorsal horns of the cervical spinal cord [3], which are only 4–5 mm apart, can be resolved by the NX due to its exquisite spatial resolution. In regions with local contrast, convergence tends to be slower at boundaries between high- and low-activity structures. Consequently, ROIs in high-contrast regions, particularly those with large surface-to-volume ratios or small volumes, are more susceptible to partial volume effects and often require additional iterations to reduce bias and resolve fine-resolution features. Moreover, compared with ^18^F, ^11^C has a substantially shorter half-life, leading to typically lower count statistics in later dynamic frames; this increases image noise and can amplify noise-induced bias. In addition, the effective sensitivity (count statistics) varies with both the tracer distribution (e.g., time-varying activity concentration across ROIs) and frame duration, so early/short frames with fewer counts can appear noisier than later/higher-count frames even under identical reconstruction settings for targeted ROIs. Accordingly, tracer- and task-specific guidance on selecting the total number of image updates in dynamic protocols warrants further investigation [45].

In this work, we performed and evaluated only one optimization approach with a 2G-PSF mixture, i.e., where all events use one PSF consisting of a mixture of two Gaussians. Further enhancement is possible with an image-domain event-specific multi-PSF approach in list-mode reconstruction for the NX scanner, i.e., using a different PSF for different subsets of the list-mode data. To date, promising preliminary results have been demonstrated using MCS data for the NX [47], [48], and future work will extend this approach to real data with full quantitative accuracy. Even so, our proposed 2G-PSF has several advantages in terms of simplicity. The multi-PSF approach requires estimating different normalization factors and mode-dependent scaling factors for sensitivity images within iterative reconstruction, increasing implementation complexity and computational cost.

Here, we did not consider spatially variant PSF modeling because the NX’s eight DOI layers, together with the typical positioning of the human brain near the center of the FOV, provide approximately uniform spatial resolution for brain reconstruction [25]. In other imaging scenarios, such as head-and-neck tumor studies [49] and carotid image-derived input function (IDIF) extraction [50], the ROI may be located at different axial positions (often closer to the axial ends of the scanner), where system resolution can differ from that in the central brain region. In future work, we will extend our PSF modeling to be spatially variant, and this extension can be applied directly to both the 2G-PSF and multi-PSF approaches.

## V. Conclusion

By characterizing reconstruction performance for detector crosstalk in a next-generation brain PET system, the NX, we quantified the resolution differences between crosstalk and noncrosstalk events. To the best of our knowledge, this is the first study to characterize and optimize detector crosstalk for reconstruction performance using both a next-generation 3-D brain phantom and real human PET data. Modeling these differences enables image reconstruction with higher contrast while preserving NX’s high sensitivity. This work provides a practical and broadly applicable recipe for future high-resolution PET systems capable of detecting detector crosstalk events, helping them achieve their full high-resolution, HR potential. Future work includes performing event-specific multi-PSF modeling to fully leverage list-mode data in which crosstalk events are identified, and incorporating spatially variant PSF modeling to further enhance image quality.

## Figures and Tables

**Fig. 1.**
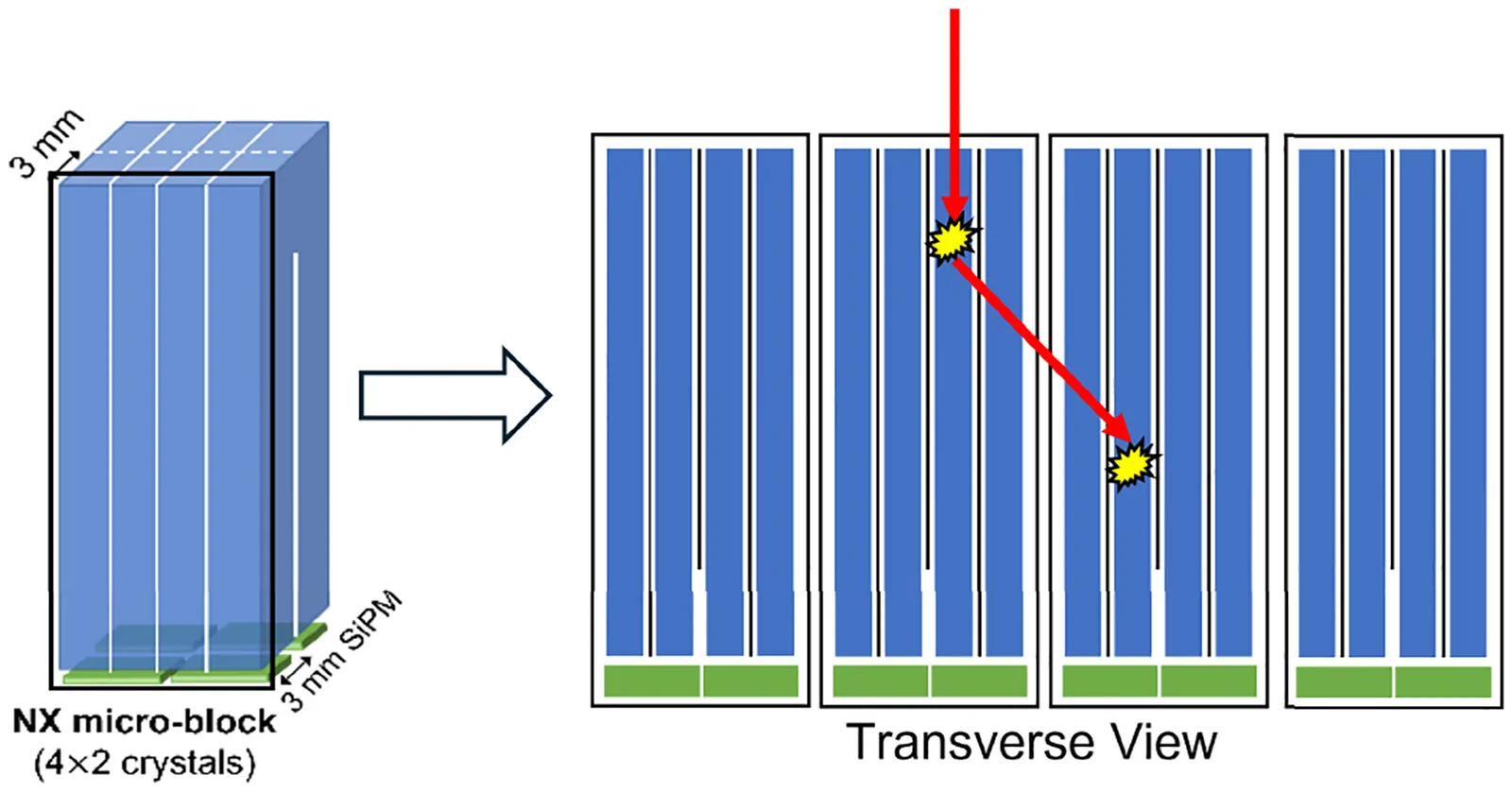
IBC in NX causes misposition of the photon interaction point.

**Fig. 2.**
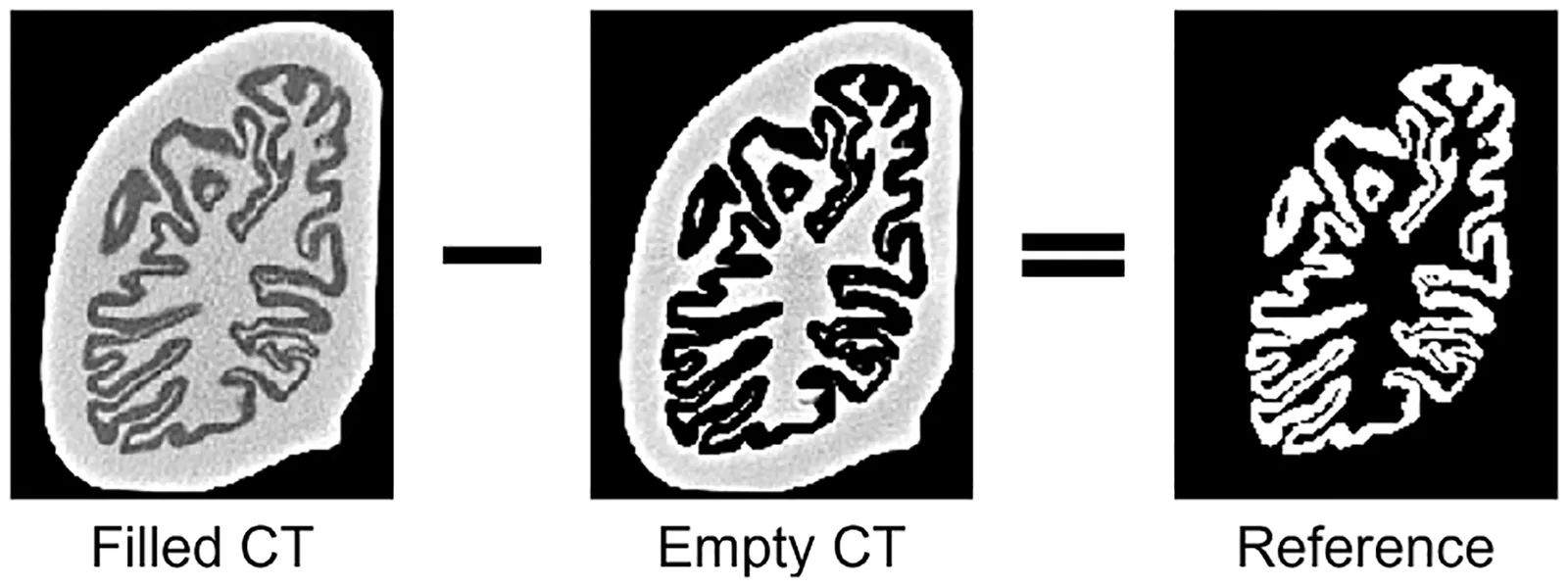
Reference image derived from high-resolution CT for HYDRA-OLG.

**Fig. 3.**
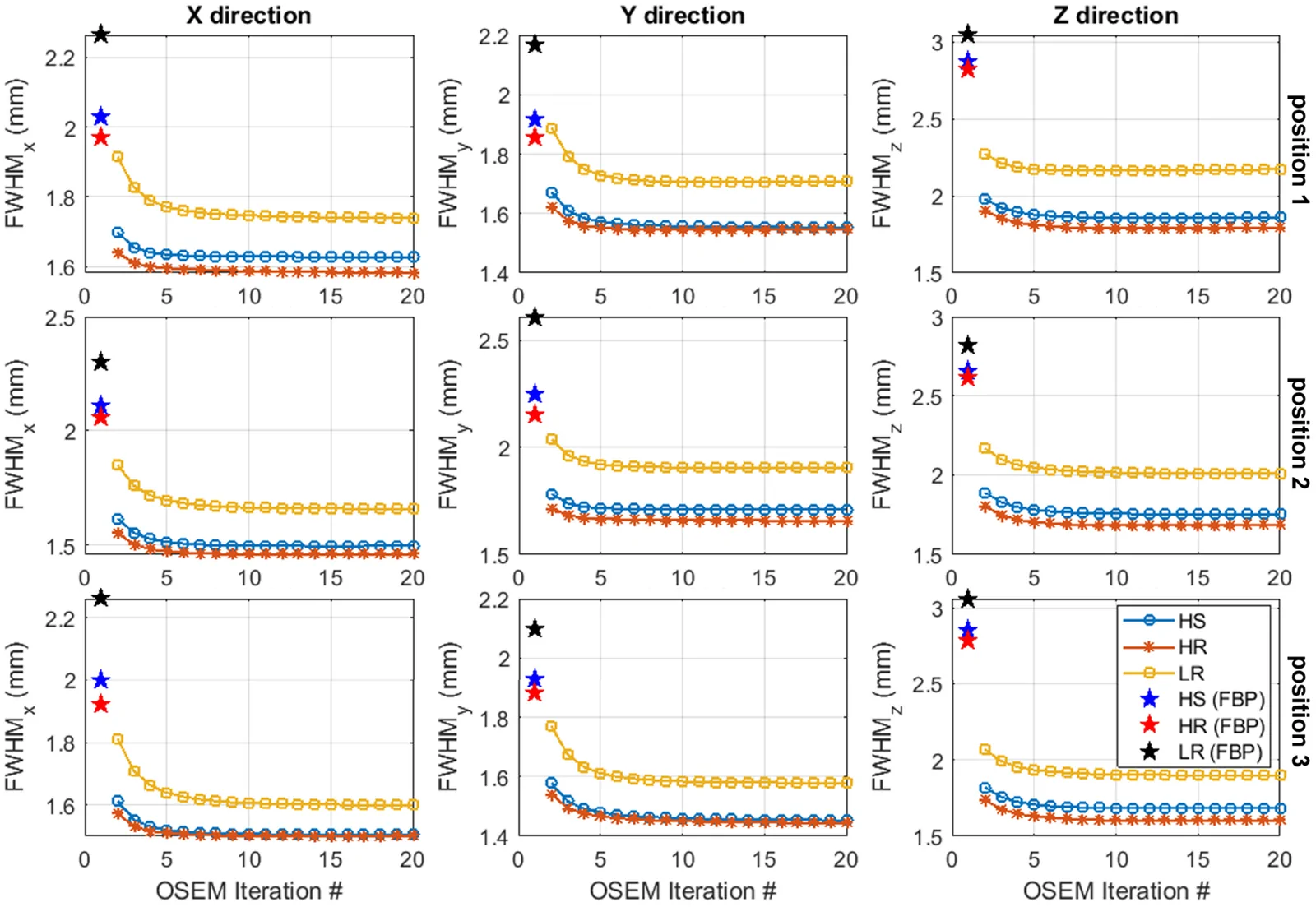
FWHM (x, y, z) from an eight-parameter 3-D-gaussian fitting for HS, HR, and LR as a function of OSEM iteration for a point source at position 1: (0, 0, 0), position 2: (0, 8 cm, 0), and position 3: (0, 0, 12 cm). FBP results are included for comparison (stars).

**Fig. 4.**
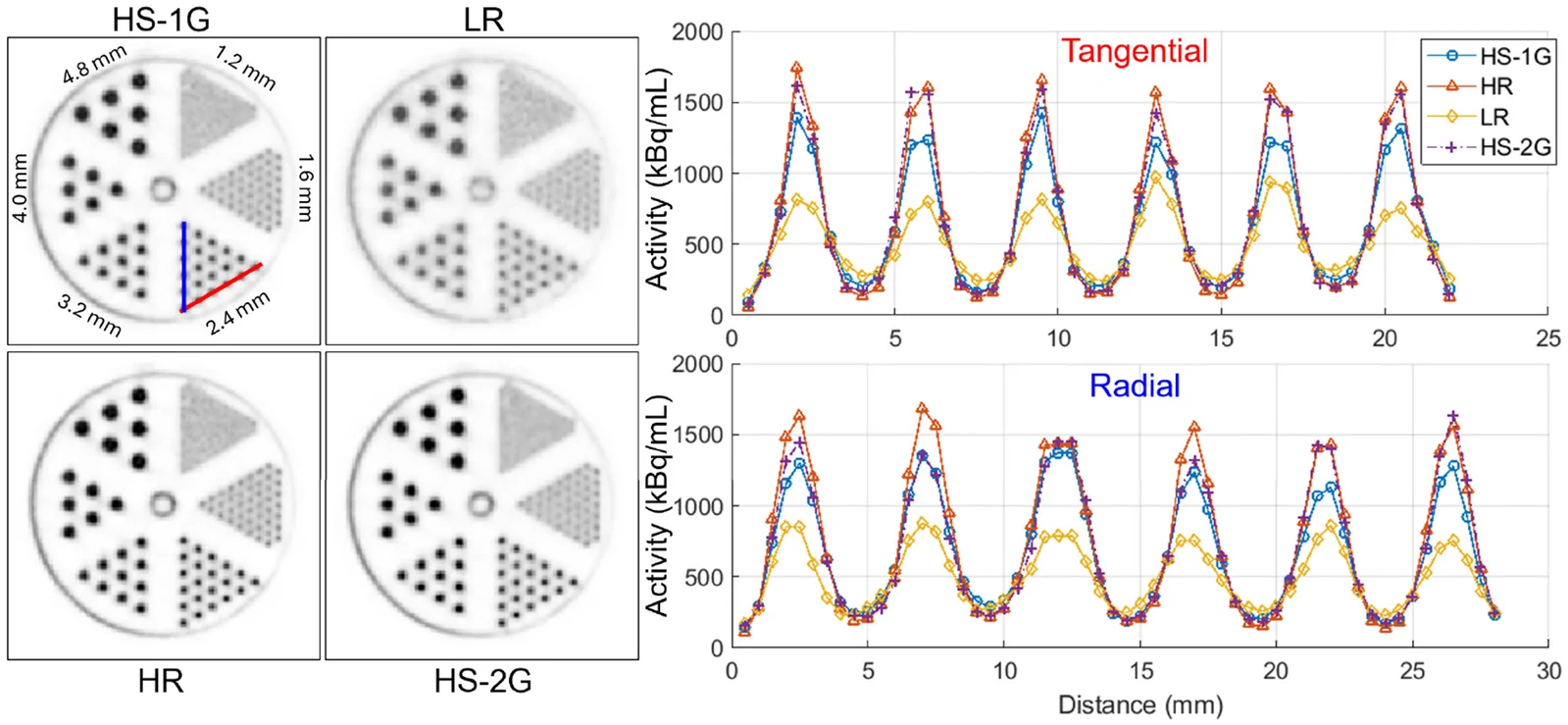
Left: OSEM reconstructions (20th iteration) for HS-1G, HR, LR, and HS-2G. Right: intensity profiles from the 2.4-mm rods in tangential (top) and radial (bottom) directions.

**Fig. 5.**
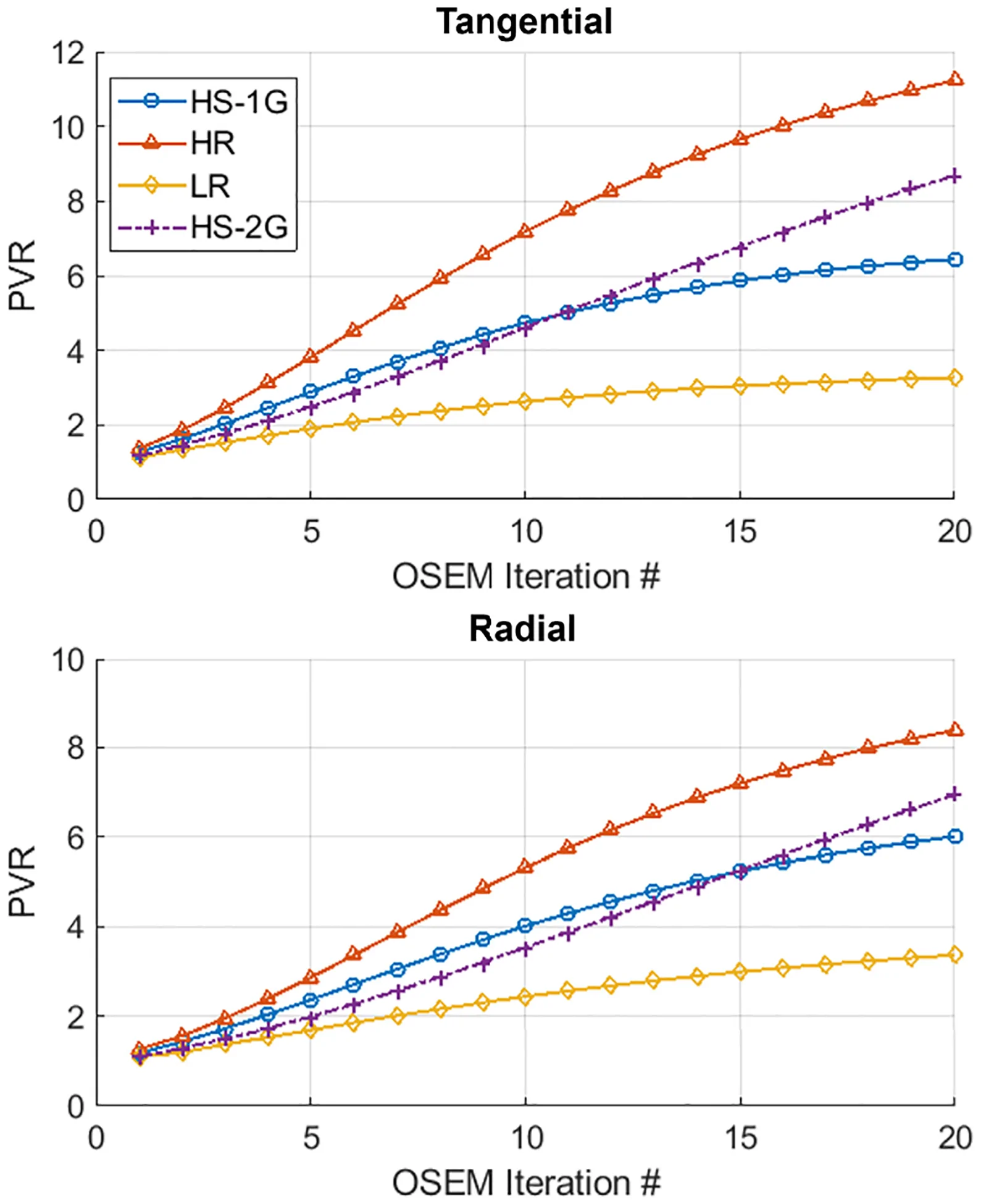
PVR as a function of OSEM iteration for 2.4-mm rods in the tangential and radial directions. HR outperforms HS-1G and LR. HS-2G approaches HR with increasing iterations.

**Fig. 6.**
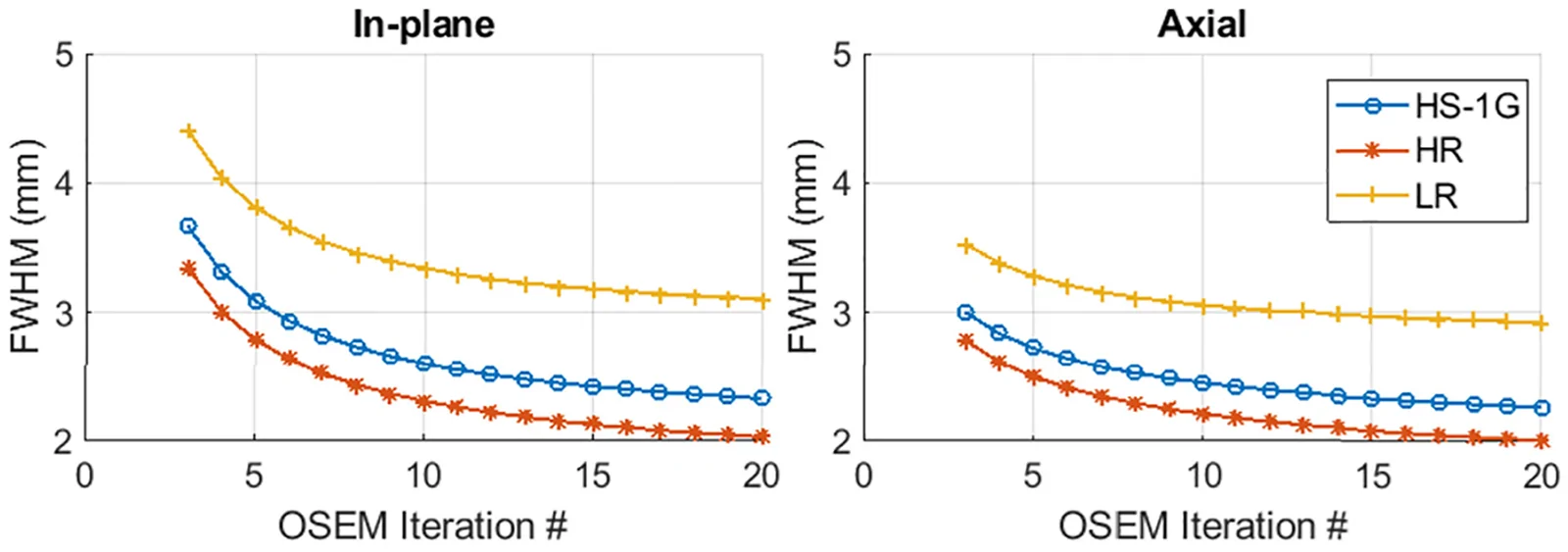
Fit Gaussian FWHM (in-plane and axial) as a function of OSEM iteration for HS, HR, and LR on the HYDRA-OLG phantom.

**Fig. 7.**
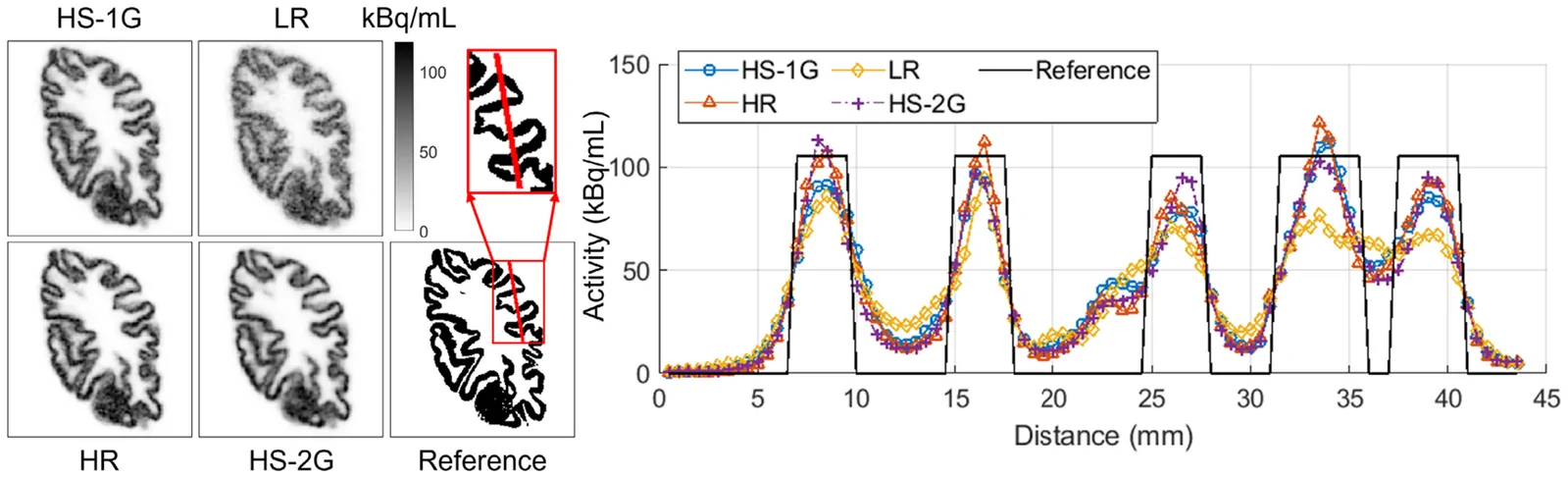
OSEM reconstructions (20th iteration) for HS-1G, HR, LR, and HS-2G with reference image. Left: representative transverse slices. Right: selected intensity profiles.

**Fig. 8.**
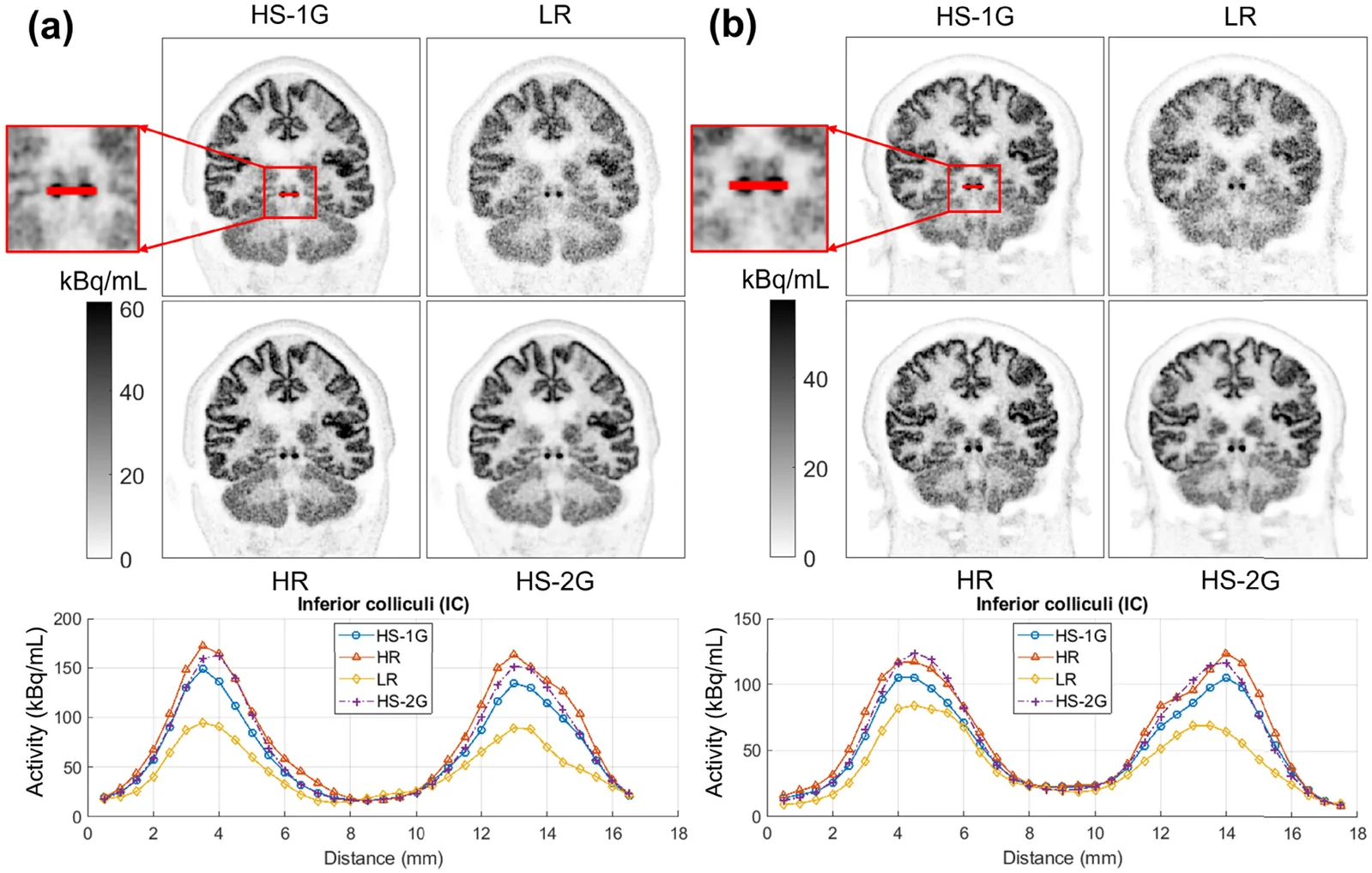
OSEM reconstructions (20th iteration) for HS-1G, HR, LR, and HS-2G of (a) and (b) two subjects imaged with 18F-FDG. Top: representative coronal slices. Bottom: intensity profiles across the IC.

**Fig. 9.**
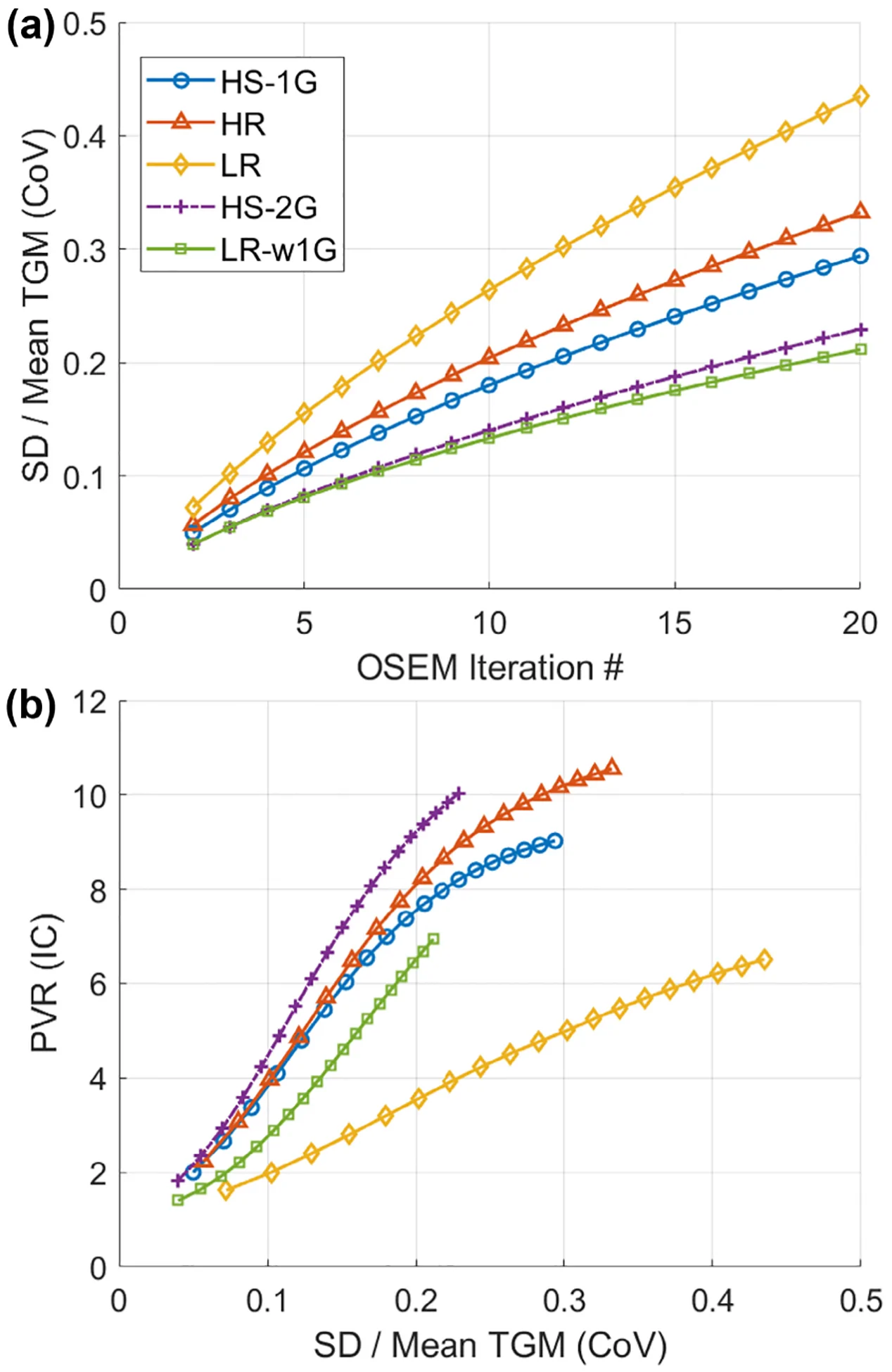
(a) CoV versus OSEM iteration and (b) PVR of IC versus CoV across OSEM iterations for HS-1G, HR, LR, HS-2G, and LR-w1G in ^18^F-FDG Subject 1.

**Fig. 10.**
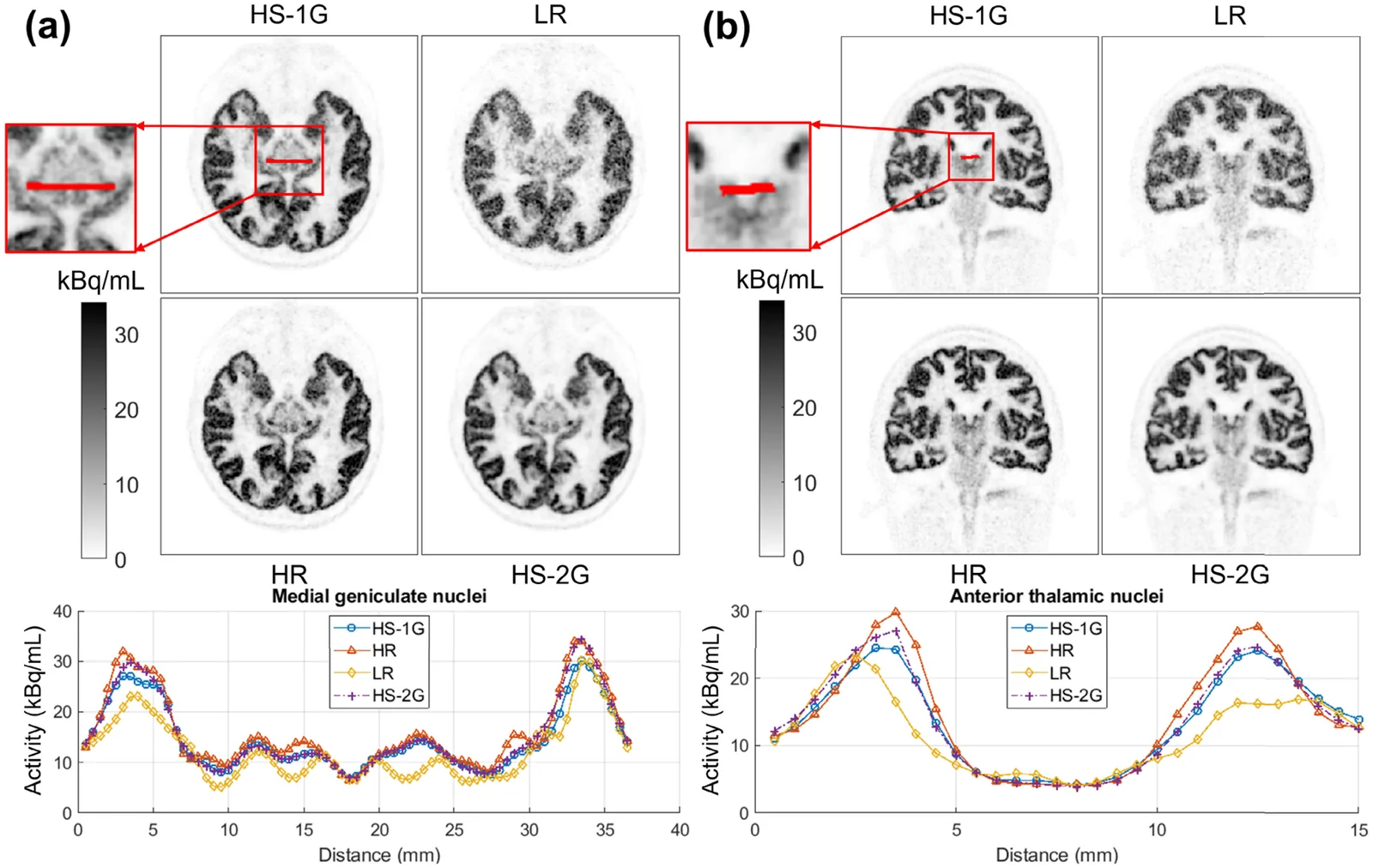
OSEM reconstructions (20th iteration) for HS-1G, HR, LR, and HS-2G of one subject imaged with ^18^F-SynVesT-1. Top: representative (a) transverse and (b) coronal slices. Bottom: intensity profiles across two distinct thalamic nuclei (left: medial geniculate nuclei; right: anterior thalamic nuclei).

**Fig. 11.**
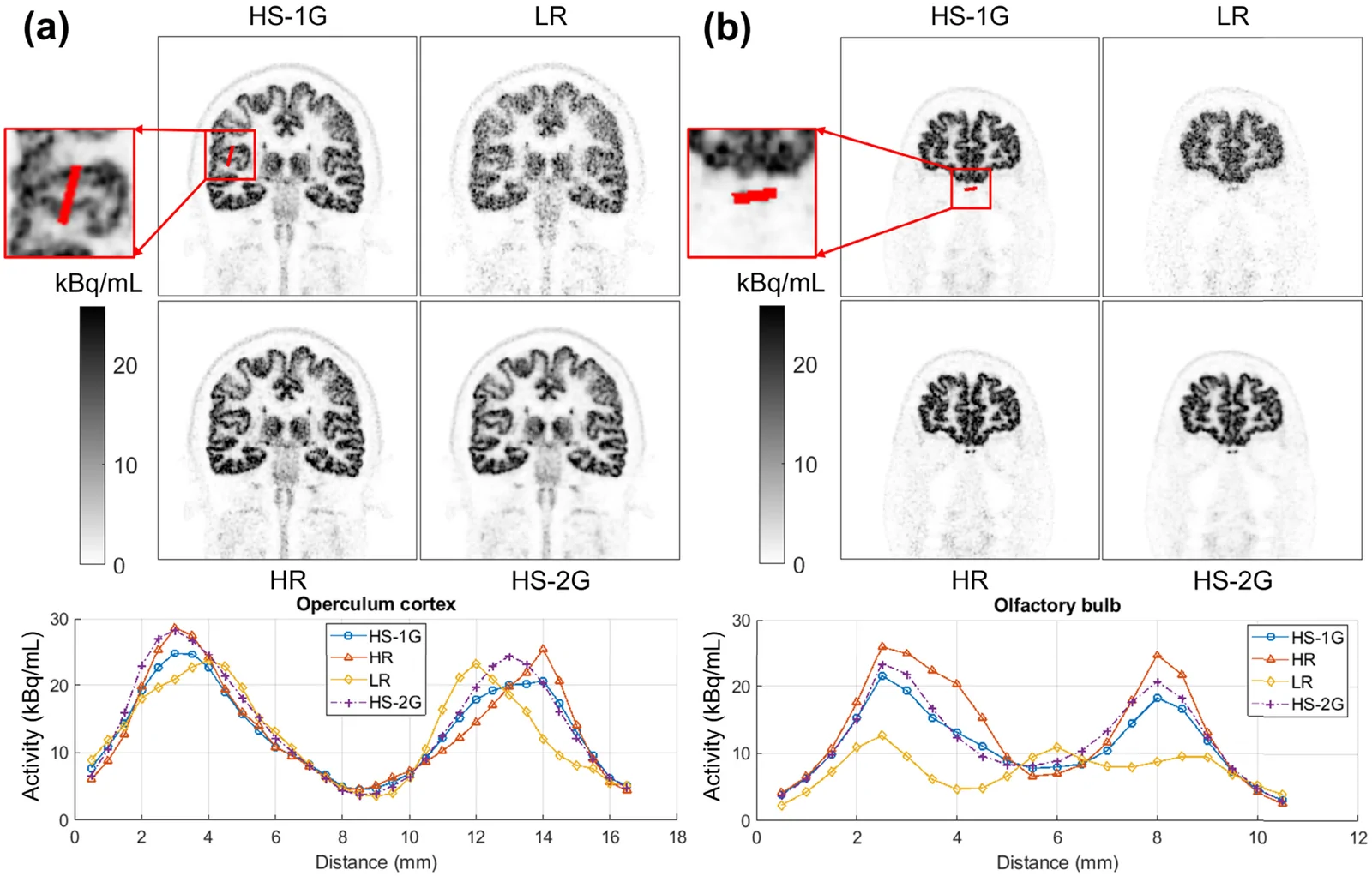
OSEM reconstructions (20th iteration) for HS-1G, HR, LR, and HS-2G of one subject imaged with ^18^F-FPEB. Top: representative (a) and (b) coronal slices. Bottom: intensity profiles across the operculum cortex (left) and olfactory bulbs (right).

**Fig. 12.**
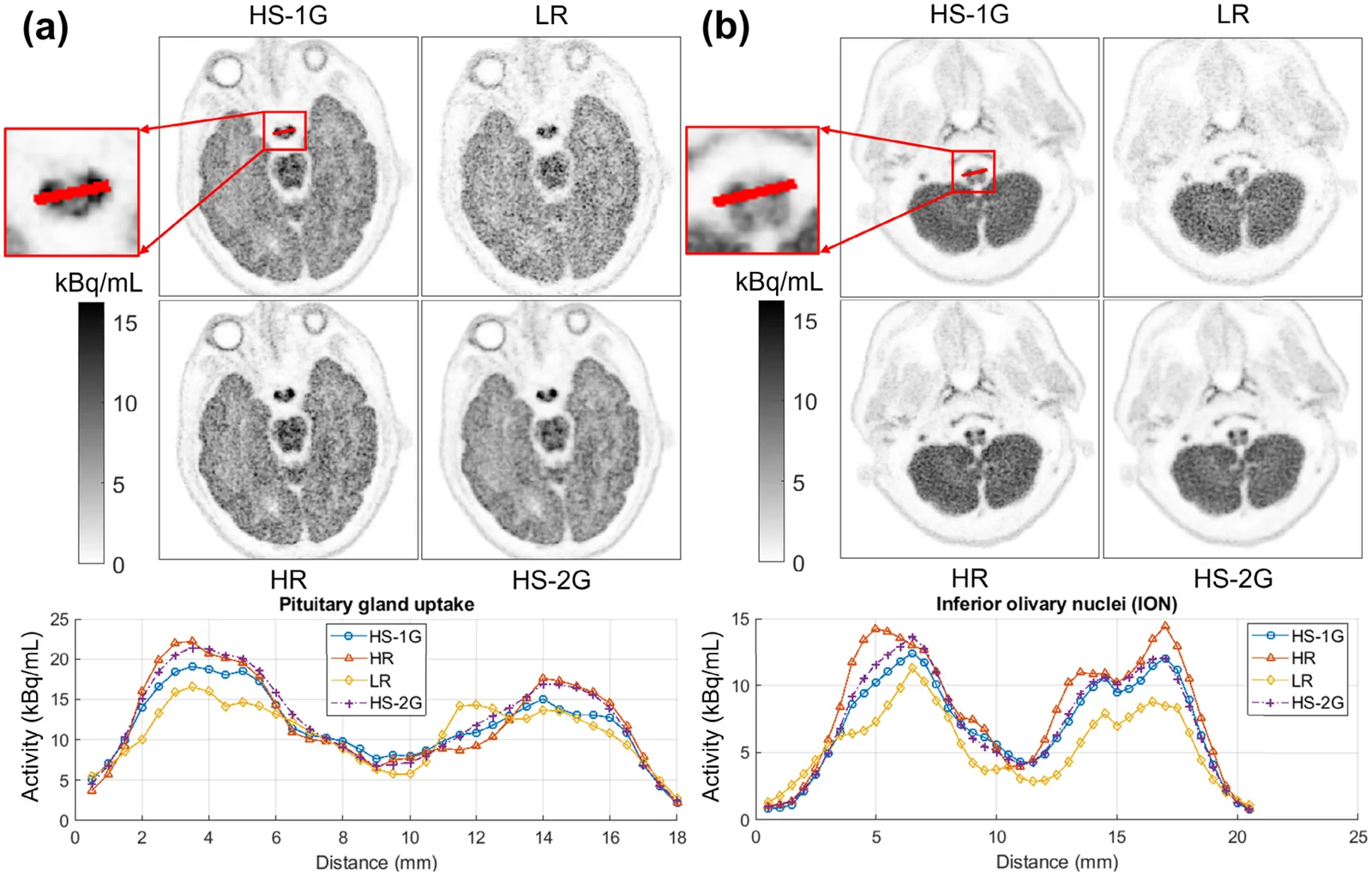
OSEM reconstructions (20th iteration) for HS-1G, HR, LR, and HS-2G of one subject imaged with ^18^F-Flubatine. Top: representative (a) and (b) transverse slices. Bottom: intensity profiles across the pituitary gland and ION.

**TABLE I:** PVR (mean ± SD) of Selected Profiles Across Subjects

	HS-1G	HR	LR	HS-2G
PVR	4.8 ± 2.2	5.7 ± 2.4	4.6 ± 1.9	5.6 ± 2.5
